# Patient perceptions of phage therapy for diabetic foot infection

**DOI:** 10.1371/journal.pone.0243947

**Published:** 2020-12-14

**Authors:** Katherine E. Macdonald, Helen J. Stacey, Gillian Harkin, Lesley M. L. Hall, Matthew J. Young, Joshua D. Jones

**Affiliations:** 1 Infection Medicine, Edinburgh Medical School: Biomedical Sciences, University of Edinburgh, Edinburgh, United Kingdom; 2 Edinburgh Medical School, University of Edinburgh, Edinburgh, United Kingdom; 3 Diabetes and Endocrinology, Queen Elizabeth University Hospital, Glasgow, United Kingdom; 4 Diabetic Foot Clinic, Outpatient Department 2, Royal Infirmary of Edinburgh, Edinburgh, United Kingdom; Monash University, AUSTRALIA

## Abstract

Infections of diabetic foot ulcers are common, generally recalcitrant and often complicated by antibiotic resistance. Alternative antimicrobial strategies are needed. Phage therapy is a promising alternative that is being rediscovered. Despite phage therapy’s 100-year history, there have been no investigations into patient thoughts and concerns. This study aimed to explore patient awareness of and concern about antibiotic resistance and gain insight into the perceptions of phage therapy among a patient group that could potentially benefit from phage therapy. Patients with an active or resolved (healed or amputated) diabetic foot ulcer were eligible to participate. A survey was distributed digitally to eligible patients across Scotland via the NHS Research Scotland Diabetes Network and hard copies were available in diabetic foot clinics at the Royal Infirmary of Edinburgh and Queen Elizabeth University Hospital, Glasgow. A focus group of five survey respondents was held in Glasgow. Fifty-five survey responses were obtained. There was a high level of awareness (76.4%; N = 55) and concern (83.3%; N = 54) about antibiotic resistance. While largely aware of viruses, most patients had not heard of phage or phage therapy. Patients were no more concerned about phage than antibiotic therapy, with most suggesting more information could alleviate any concerns. Patient acceptability of phage therapy was high, a finding confirmed by the focus group. Patients are concerned about antibiotic resistance and supportive of ‘new’ antimicrobials. We have demonstrated that patients are supportive, enthusiastic and accepting of phage therapy. Although ‘Western’ phage therapy remains in its infancy, an understanding of patient ideas, concerns and expectations will be important in eventually shaping and successfully reintroducing phage therapy.

## Introduction

Globally, in 2019 there were an estimated 463 million adults living with diabetes mellitus, herein diabetes, with the global prevalence projected to rise to 700 million adults by 2045 [1]. Its estimated that around one third of diabetic patients will develop a diabetic foot ulcer (DFU), a complication of poorly controlled diabetes, and that over half of such ulcers will become infected [2,3]. Infected diabetic foot ulcers, termed diabetic foot infections (DFIs), lead to amputation in approximately 20% of moderate or severe cases [2].

A diverse range of microorganisms may be isolated from DFIs, with Gram positive bacteria, particularly *Staphylococci*, most commonly isolated [4]. Antibiotic resistance is common, for example, the prevalence of methicillin resistant *Staphylococcus aureus* in DFIs is considered to be 15–30% [5,6], and the prevalence of vancomycin resistant *Enterococci* has been reported at 7.1% and observed to be 10.5% in local clinical audit data [7,8].

Wound care and antibiotics are the mainstay of DFI treatment. The chronic and recalcitrant nature of these, often antibiotic resistant, infections typically requires the administration of lengthy courses of antibiotics. These extended courses of antibiotics place patients at risk of antibiotic-associated side effects, such as *Clostridium difficile* infection, and may themselves contribute to the development of antibiotic resistance. Moreover, the efficacy of haematogenously distributed antibiotics may be hampered by peripheral arterial disease, a common comorbidity among DFI patients [9]. The combination of the antibiotic resistance crisis and the significant clinical and financial impact of DFIs on patients and the broader healthcare system make it imperative to explore alternative therapeutic strategies.

Bacteriophage (phage) therapy is a promising antimicrobial strategy that is being rediscovered across Western medicine [10]. Phage therapy is increasingly being used compassionately and in clinical trials across Europe and the US without notable side effects [11–14]. Phage are naturally-occurring viruses of bacteria that were discovered in the UK in 1915 and have been used to treat infection somewhere in the world since 1919, largely in the geopolitical East [15]. There is an extremely large and diverse population of naturally occurring phage, estimated at 10^31^ phage on the planet, that may be found wherever their bacterial hosts exist [16]. The use of phage to treat bacterial infection, known as phage therapy, was widespread in the West in the 1920s and 30s, but became redundant upon the discovery of antibiotics, partly as susceptibility screening was not required prior to antibiotic use. Antibiotic resistance has driven renewed interest in phage therapy.

The prevalence, morbidity, mortality and antibiotic challenges associated with DFIs make them a prime clinical indication for phage therapy. Indeed, phage therapy has successfully been used to treat DFIs and other chronic wounds [17–20]. However, the successful reintroduction of phage therapy into Western medicine will require an understanding of patient ideas, concerns and expectations around phage therapy. Naturally, with phage therapy, such patient-centred issues may be very different compared to those associated with traditional chemotherapeutics.

To the best of our knowledge, in over 100 years of phage therapy, there have been no investigations into patient perceptions and concerns regarding phage therapy. One recent study asked patients about their satisfaction with the outcome of phage therapy but did not engage patients regarding their broader perceptions and concerns [21]. To address this, we undertook the first ever patient perception survey of phage therapy. Working with the NHS Research Scotland Diabetes Network, we conducted a nationwide survey among Scottish DFU patients with an active or resolved (healed or amputated) diabetic foot ulcer. The objectives of this study were to gauge diabetic foot patient perceptions of, and receptivity to, phage therapy. To help place patient perceptions about phage therapy in context, we first assessed patient’s awareness and concern about antibiotic resistance. Subsequent questions explored patient’s awareness and concerns surrounding phage therapy and how such concerns could be mitigated. The nationwide survey was supported by a focus group held in Glasgow.

## Methods

### Survey design

An anonymous five-section survey was designed (S1 File). The survey elicited quantitative and qualitative responses, using a mixture of tick-box, rating scale and free-text responses. Briefly, section one collected demographic data and information about past patient exposure to antibiotics. Section two gauged patient awareness of the antimicrobial resistance crisis, associated concerns and further actions patients felt should be taken to address the crisis. Section three assessed patient awareness of phage and phage therapy. Reasoning that most patients would be unfamiliar with phage therapy, section four began with some background information about phage and phage therapy, before assessing patient concerns about phage therapy, including relative to antibiotics, and how those concerns might be mitigated. A longer debrief section followed section four, with some basic information about what phage are, the history of phage therapy, how phage therapy works and some of the challenges associated with phage therapy. In section five patient acceptability of phage therapy was explored and patients were given the opportunity to make any further comments. Section five also contained an option for patients to provide an e-mail address to receive the results of the study. Every survey was given an anonymous unique identifier.

The survey was accompanied by both an approved participant information sheet (PIS) and a data protection information sheet (S2 File), completed using templates provided by the Academic and Clinical Central Office for Research and Development (NHS Lothian). The PIS contained details of an independent contact, should patients have wanted to discuss the study with someone beyond the direct study team. The survey was reviewed by NHS diabetologists and phage experts. The diabetes Public and Patient Interaction groups in Dundee and Edinburgh also reviewed the survey. Before distribution, the survey was piloted with a small number of DFI patients and subsequently refined further. The pilot survey only evaluated the practical (e.g. layout, font size) and English-language suitability of the survey, no data was collected.

### Survey distribution

During autumn 2019 the survey was made available to DFU patients across Scotland in the English language and in a digital format. This was supplemented by the placement of hard copies in diabetic foot clinics at the Edinburgh Royal Infirmary (ERI) and Queen Elizabeth University Hospital (QEUH) in Glasgow. Both the paper and digital copies were advertised using approved leaflets and posters in clinics. The NHS Research Scotland Diabetes Network (NHS RSDN) holds a national register of diabetic patients that have consented to being informed about research opportunities. The Research Network assisted in distributing the digital survey link to eligible registered patients across Scotland by electronic and paper invitations. The platform onlinesurveys.ac.uk was used to host the digital survey, with institutional access provided by the University of Edinburgh.

Eligible patients had either an active diabetic foot ulcer or a resolved past ulcer (healed or amputated). Eligible patients were also NHS RSDN registered patients or patients from the diabetic foot clinics at ERI or QEUH, aged 18 years or over, and confident communicating in English.

Patients were able to self-select into the study by choosing to complete the survey as a paper copy in clinic or online. They were not obligated to complete the survey and consent was implied by return of the survey. There was an eight-week window for the receipt of survey responses.

### Focus group

Only survey respondents were eligible to attend the focus group sessions. Patients were able to self-select to attend the focus groups by responding to an advert. The PIS for the focus group session and informed consent form were enclosed with both formats of the survey (S3 File). Additionally, members of the NHS direct care teams at each clinic were able to approach patients completing the survey in clinic. Four focus group sessions had been planned, but there was only sufficient demand to hold one, which took place towards the end of the survey window at the QEUH in Glasgow.

### Data interpretation and processing

Hard-copy survey responses were entered into a password protected Microsoft Excel spreadsheet. Two authors adjudicated where responses to quantitative questions were ambiguous, there were five such responses. Prefixes or suffixes to numerical values were discarded in three cases, for example ‘8+’ was entered as 8, ‘16+’ as 16 and ‘around 48’ was entered as 48. In one case a text-based response to a quantitative question was converted into a numerical value; ‘once weekly’ was transcribed as 52. A numerical range was given by one patient; to achieve one figure the rounded average of the range was taken as the response value. Responses in which the handwriting was unclear were checked by another author. One hard-copy respondent made comments outside any defined response box, and these were disregarded in subsequent per-question analyses. Digital survey responses were downloaded and entered into the same spreadsheet. Microsoft Excel was used to perform descriptive statistics and RStudio (Mac, Version 1.1.456) was used to perform the Chi Square Test of Independence and 2-sample test for equality of proportions, both with Yate’s correction for continuity. NVivo software (QSR International Pty Ltd. Version 11.4.3, 2015) was used to facilitate qualitative manifest analysis of survey responses on a per question basis. The broad themes identified among survey responses were reviewed by two authors (KEM, JDJ). In some cases, the frequency with which themes were observed among the survey responses (n) was greater than the sample population for that question (N).

### Approvals

This study was approved by the London—Surrey Borders Research Ethics Committee (19/LO/0307). NHS Lothian (2019/0028) and NHS Greater Glasgow and Clyde (GN19DI055) research and development offices also approved the study. Consent to participate in the survey was informed by the provision of an approved participant information sheet, with consent to participate implied by return of the survey. Consent to participate in the focus group was informed by the provision of an approved participant information sheet, with consent to participate implied by attendance at the focus group, where written consent was obtained.

## Results

### Survey responses

A total of 402 patients on the NHS RSDN research register that met the survey eligibility criteria were invited to take part, representing 354 postal and 48 e-mail invitations. The response rate of NHS RSDN registrants was 5.0% (N = 20). A response rate of patients engaged by diabetic foot clinics, e.g. who completed a paper copy or responded to the leaflet or poster, was not calculable. However, a total of five patients from the Royal Infirmary of Edinburgh (RIE) and one from the Queen Elizabeth University Hospital (QEUH) completed the digital survey. This was supplemented by seven paper hard-copy responses from RIE and 22 from QEUH. A total of 55 digital and hard-copy responses was obtained (S4 File). Not every respondent answered every question, therefore the frequency of responses to each question will be indicated throughout (N) and distinguished from frequencies quoted as part of data analysis (n).

### Sample population

Section one of the survey collected anonymous demographic data about the respondents (Table 1). Fifty-five patients completed the survey, with the study population comprising 22 females and 33 males. The age range of respondents was 35 to 82 years, with a mean of 57.4 ± 11.0. The study population was very familiar with antibiotic chemotherapy, with almost all (98.2%) respondents (N = 55) having taken antibiotics for any illness, while 1.8% didn’t know. The respondents (N = 53) had taken on average 3.5 courses of antibiotics for any illness in the 12 months prior to undertaking this survey, with a range of zero to 15 courses. The study population was also regularly engaged with the healthcare system, having been an outpatient or inpatient for any reason in the 12 months prior to the survey an average of 16.2 times (N = 54), with up to 107 hospital contacts in one case.

**Table 1:** Respondent demographics. Data are shown as mean ± Standard Deviation (SD).

**Age** N = 55	57.4 ± 11.0	
**Gender** N = 55	Female	40%
	Male	60%
**Previously had antibiotics** N = 55	Yes	54
	No	0
	Don’t know	1
**Courses of antibiotics in last 12 months** N = 53	Mean ± SD	3.5 ± 2.9
	Mode	2
	Range	15 (0–15)
**Frequency of inpatient and outpatient visits for any reason in last 12 months** N = 54	Mean ± SD	16.5 ± 21.6
	Mode	3
	Range	107 (0–107)

### Antibiotic resistance: Awareness and concerns

The patients showed a high level of awareness of antibiotic resistance (N = 55), with 76.4% (n = 42) having heard of antibiotic resistance prior to this survey. The patients that had heard of antibiotic resistance were asked to state where they had heard of it. The responses from the 42 such patients fell into six broad categories. Most patients cited media sources (59.5%; n = 25), interaction with a healthcare setting (33.3%; n = 14), their own education (14.3%; n = 6) or experiences (e.g. of having a resistant infection; 4.8%; n = 2). Three patients cited ‘various sources’ (7.1%) and one responded ‘not heard of it?’ (2.4%). When those who had previously heard of antibiotic resistance (N = 41) were asked what they thought the causes were, seven themes were identified on 52 occasions. Most responses cited inappropriate use of antibiotics (65.9%; n = 27), primarily ‘overuse’. A change in the bacteria, such as ‘mutation’ or ‘resistance’ was cited by 24.4% (n = 10). Partial completion of a course (12.2%; n = 5), insufficient antibiotics (7.3%; n = 3) and overuse in agriculture (4.9%; n = 2) were other reasons given. Three patients (7.3%; n = 3) suggested that the cause of resistance was the human ‘body [becoming] immune to antibiotics’. There were two responses in which a theme could not be identified (4.9%): ‘not sure’ and ‘not heard of it?’.

**Fig 1:**
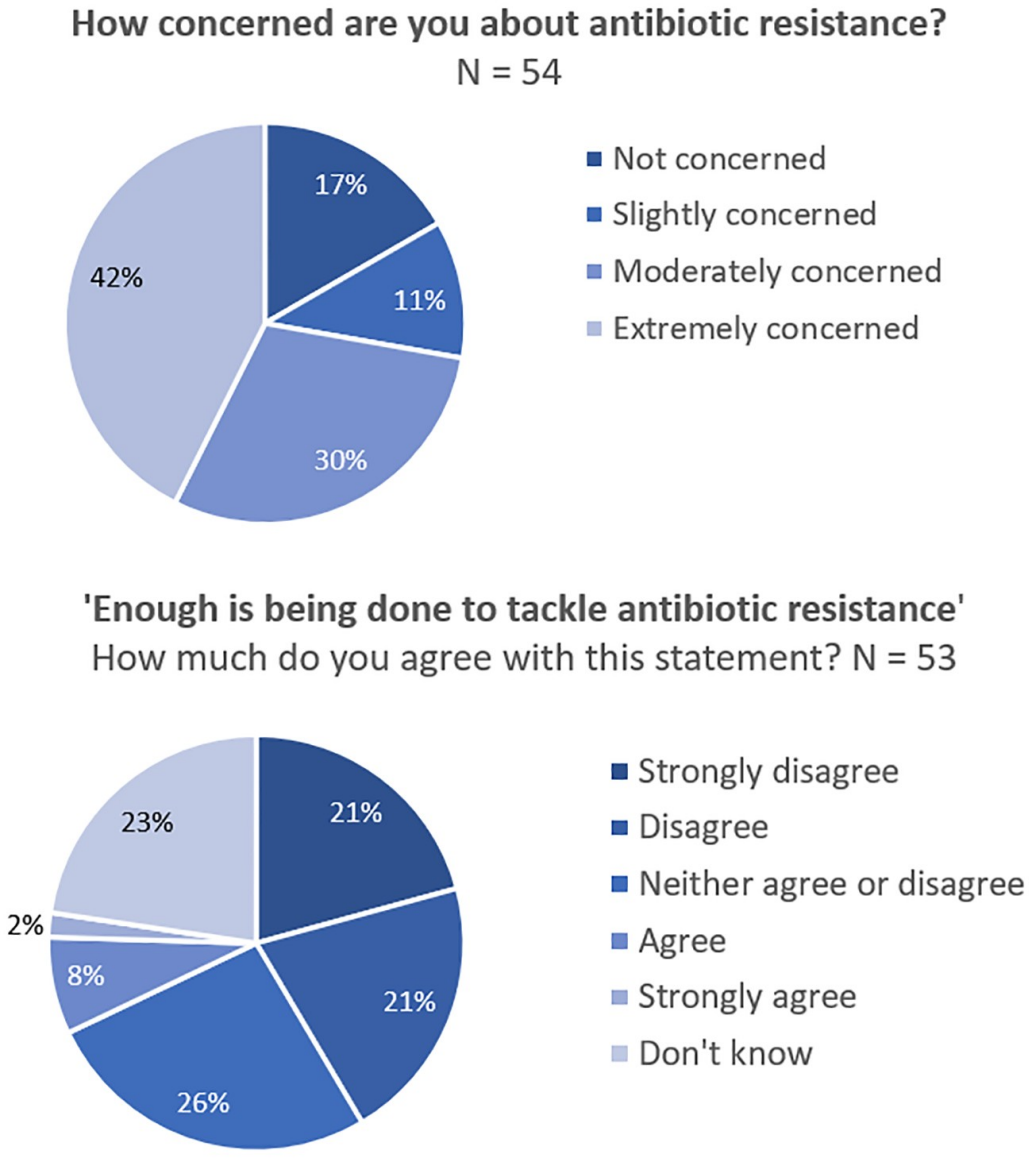
Antibiotic resistance: Awareness and concerns. (A) Patient concern about antibiotic resistance, N = 54. (B) Patient evaluation of tackling antibiotic resistance, N = 53.

There was a high level of concern among patients about antibiotic resistance, with 72.2% ‘extremely’ (42.6%) or ‘moderately’ (29.6%) concerned. A further 11.1% were ‘slightly concerned’ and 16.7% were ‘not concerned’ about antibiotic resistance (N = 54; Fig 1a). When presented with the statement ‘enough is being done to tackle antibiotic resistance’, 42% of respondents either ‘strongly disagreed’ (20.8%) or disagreed (20.8%; N = 53; Fig 1b). Around one quarter (26.4%) neither agreed or disagreed, and 22.6% didn’t know. Just 7.5% of respondents agreed that enough was being done to tackle antibiotic resistance, and only 1.9% strongly agreed. The patients that suggested that more should be done to tackle antibiotic resistance were asked to elaborate. Comments were provided by 29 patients and broadly fell into seven themes. Most patients (34.5%; n = 10) said more research (explicitly or implicitly into antibiotics) was needed. An equal number of patients said that they felt more research into alternative approaches was needed (34.5%; n = 10). Reduced or better targeted antibiotic prescriptions (24.1%; n = 7), patient education (20.7%; n = 6) and reduced pressure to prescribe (6.9%; n = 2) or use in agriculture (6.9%; n = 2) were also suggested as strategies to tackle antibiotic resistance. Three patients wrote ‘don’t know’ (10.3%). Antibiotic resistance aside, 96.4% of patients (N = 55) thought that alternatives to antibiotics should be investigated, with just 3.6% disagreeing.

### Awareness of viruses and phage

The patients had a high level of awareness of viruses (N = 55), with 76.4% (n = 42) having heard of viruses, compared to 20% (n = 11) who had not prior to this survey; 3.6% (n = 2) were ‘not sure’. Note that this survey was conducted in late 2019, before the COVID-19 pandemic.

However, among those that were aware of viruses, awareness of phage was poor. Of those who had previously heard of viruses (N = 42), just 23.8% (n = 10) had heard of ‘viruses that kill bacteria, also known as ‘bacteriophage’ or ‘phage”; 71.4% (n = 30) had not heard of phage and 4.8% (n = 2) were not sure. The 10 patients that had heard of phage cited 11 sources. Six cited media sources (four of which cited online media), two cited their own education, one cited discussions with healthcare professionals and one reported ‘various sources’. There was one unclassified response (‘no’).

Patient awareness of phage therapy was scant (N = 55), with only 9.1% having heard of phage therapy before this survey, compared to 87.3% that had not and the 3.6% that were unsure. Those who had heard of phage therapy (n = 5) had done so through six sources including media (n = 4), with one individual citing a television program about the use of phage therapy in Russia. Other responses cited ‘discussions with various healthcare professionals’ (n = 1) or ‘various sources’ (n = 1).

### Patient concerns about antibiotic or phage therapy

**Fig 2:**
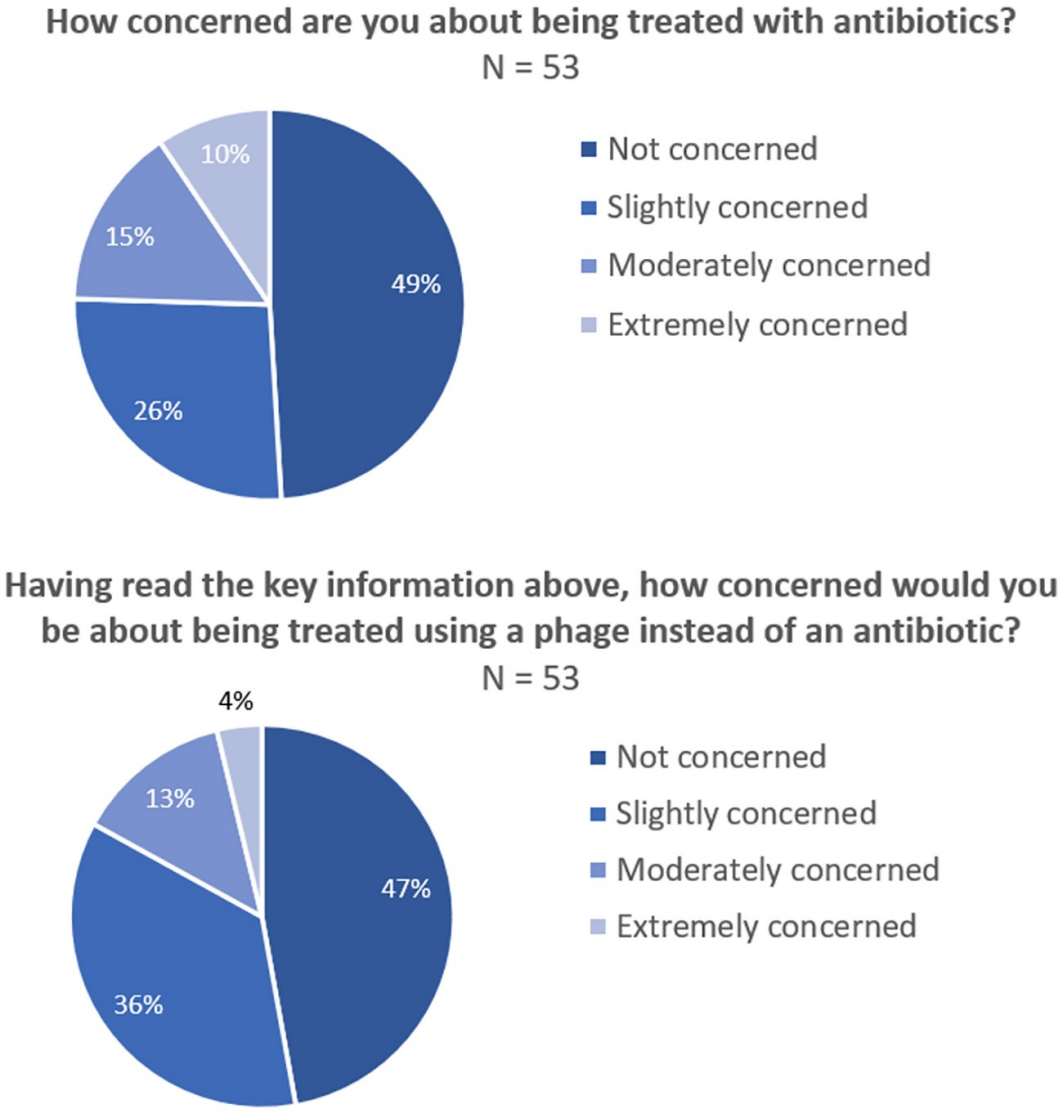
Patient concern about antibiotic or phage therapy. (A) Patient concern about antibiotic therapy, N = 53. (B) Patient concern about phage therapy, N = 53.

Having been presented with five simple key points about phage therapy, section four used identical four-part rating scales to comparatively evaluate patient’s potential concerns about antibiotic or phage therapy. Somewhat surprisingly just 49.1% of patients were ‘not concerned’ about being treated with antibiotics (N = 53). Just over one quarter of patients were slightly concerned about antibiotic therapy (26.4%), while 15.1% were ‘moderately concerned’ and 9.4% were ‘extremely concerned’ (Fig 2A). When the same question was asked about phage therapy (N = 53), the number of patients ‘not concerned’ fell by 2% to 47.2%. Among those patients who were concerned, the level of concern was less than that expressed about antibiotic therapy with 35.8% ‘slightly concerned’, 13.2% ‘moderately concerned’ and only 3.8% ‘extremely concerned’ (Fig 2b). A non-parametric 2-sample test for equality of proportions with Yate’s correction for continuity was used to compare the proportions of ‘extremely concerned’ patient responses to antibiotic (9.4%) versus phage (3.8%) therapy. A p value of 0.1704 was obtained, demonstrating no significant difference in the proportions of ‘extremely concerned’ individuals between the treatments.

We used a Chi Square Test of Independence with Yate’s correction for continuity to compare the overall levels of concern expressed across all categories, surrounding antibiotic and phage therapy. The null hypothesis was that ‘there is no difference between patient concern when using antibiotics versus phage in the study cohort’. A p value of 0.546 was obtained, however, possible p value uncertainty was reported. To ensure an accurate Chi Square approximation, the test was repeated with simulated data where a comparable value of 0.5652 was obtained. Reassuringly, both p values were markedly larger than the <0.05 threshold for statistical significance, confirming that there was no difference in overall patient concern between the two antimicrobial strategies. The low level of concern about phage therapy was again reflected when patients were asked if they would consider phage therapy if they had an infection for which no antibiotic was available (N = 55). The majority, 89.1% of participants said they would consider phage therapy in such circumstances, with 9.1% not sure and 1.8% saying they would not.

A free-text space was provided for patient to express any concerns about phage therapy and the responses were divided into nine themes (Table 2). Most of the 43 patients who responded stated no concerns about phage therapy (44.2%; n = 19). Areas of concern included the safety (23.3%; n = 10) and efficacy (vs. antibiotics; 20.9%; n = 9) of phage therapy, side effects (14.0%; n = 6) and queries about how phage could be administered (4.7%; n = 2). Other responses requested more information (4.7%; n = 2), asked why phage therapy wasn’t already used (4.7%; n = 2), stated ‘don’t know’ (2.3%; n = 1) or were uninterpretable (2.3%; n = 1). When asked what could be done to reduce their concerns, most of the 38 respondents expressed a desire for more information (39.5%; n = 15) or research (23.7%; n = 9). Some patients reiterated their absence of concerns (18.4%; n = 7). Other patients said they would like to hear about the experiences of other patients who had received phage therapy (13.2%; n = 5). The remainder said they would like professional assurance (e.g. from a doctor; 5.3%; n = 2), didn’t know (5.3%; n = 2) or gave responses that could not be classified (5.3%; n = 2; ‘no’, ‘even better’).

**Table 2:** Themes identified among concerns about phage therapy, N = 43.

Concern	% of patients	n
No concerns	44.2	19
Safety	23.3	10
Efficacy (vs. antibiotics)	20.9	9
Side effects	14.0	6
More information	4.7	2
Why not already used?	4.7	2
Administration	4.7	2
Don’t know	2.3	1
Uninterpretable	2.3	1

### Patient acceptability of phage therapy

**Fig 3:**
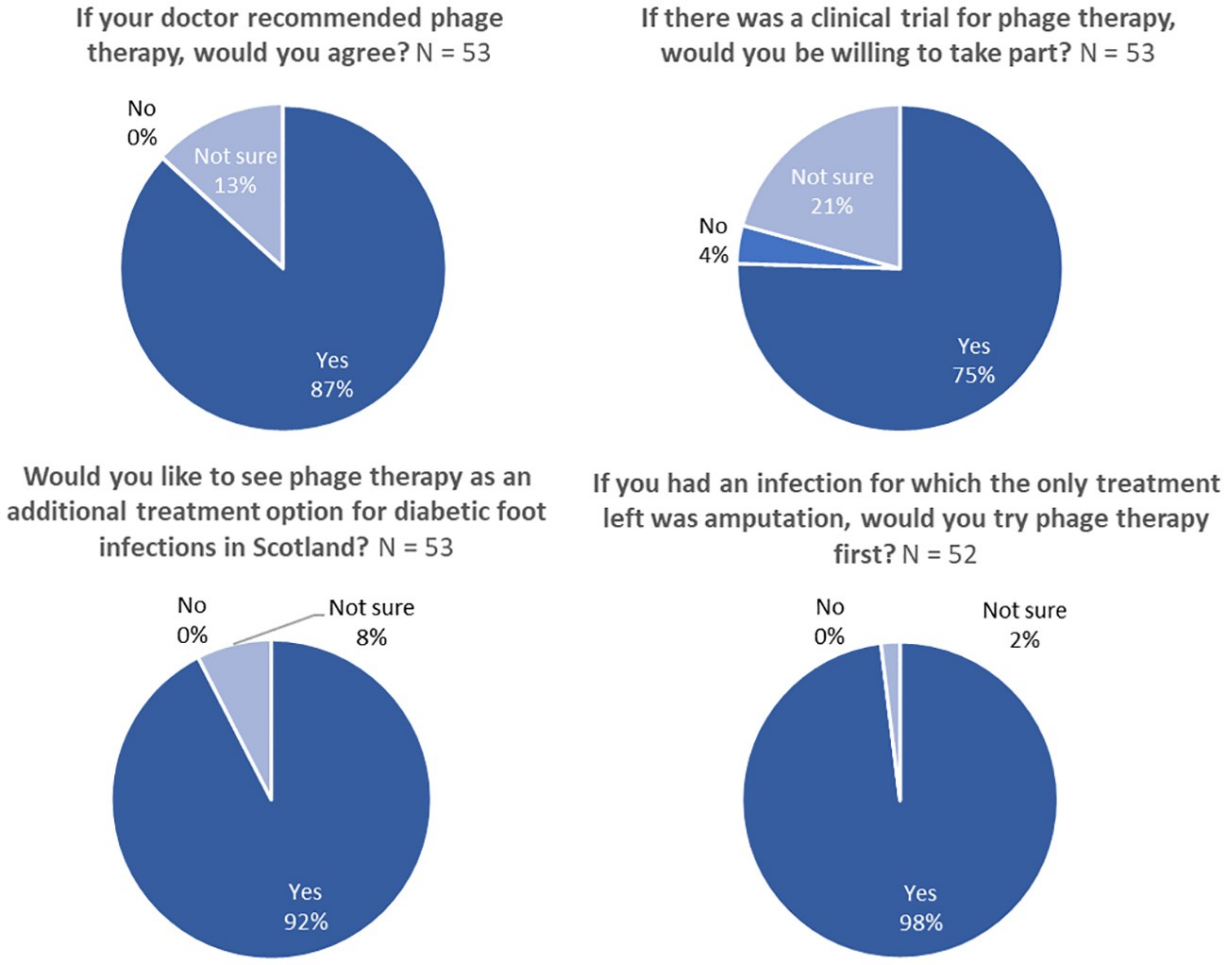
Patient acceptability of phage therapy. (A) Acceptability of phage therapy if recommended by a doctor, N = 53. (B) Acceptability of phage therapy as an alternative to amputation, N = 52. (C) Appetite to participate in a clinical trial of phage therapy, N = 53. (D) Demand for phage therapy to be an additional treatment option in Scotland, N = 53.

A debrief section followed section four of the survey and provided simple factual information about phage, the history of phage therapy, how phage therapy works and some challenges faced by phage therapy. Having read the debrief, the patients were asked about their willingness to receive phage therapy. An overwhelming majority said they would accept phage therapy if it was recommended by their doctor (86.8%; Fig 3A), with none refusing and 13.2% ‘not sure’ (N = 53; Fig 3A). Of the seven participants who were not sure, five provided explanation: four wanted more information about phage and one was concerned that phage may not be as effective as antibiotics. When asked whether they would consider phage therapy as an alternative to amputation if there was no other treatment option, all but one patient (98%) said they would try phage therapy first (Fig 3B). The respondent who was ‘not sure’ then commented that they ‘would try anything’; suggesting that they may not have fully understood the question and that in fact 100% of respondents were willing to try phage therapy as an alternative to amputation.

There was substantial appetite to participate in a clinical trial of phage therapy with 75.5% (n = 40) expressing a willingness to take part (Fig 3C). Only 3.8% (n = 2) would be unwilling to participate, with 20.8% (n = 11) ‘not sure’. Seven of the eleven unsure respondents left a comment explaining their decision. Four requested more information on phage, two would be uncomfortable being trial subjects, and the remainder expressed concerns about the trial location (‘would depend on where trials were held’; n = 1) or would need support from a medical professional before taking part (‘would prefer to be advised by my physician’; n = 1).

Regarding the future of phage therapy, an overwhelming majority of patients (92.5%; n = 49) said that they would like to see phage therapy as an additional treatment option for DFIs in Scotland (N = 53; Fig 3D). Of the 7.5% (n = 4) who were ‘not sure’, three provided further written responses. They expressed concerns about phage safety and efficacy (66.7%, n = 2), or provided a response which could not be classified (33.3%, n = 1, ‘new to me’). Overall, these data show an exceptional appetite for phage therapy among DFI patients in Scotland.

### Additional comments

**Fig 4:**
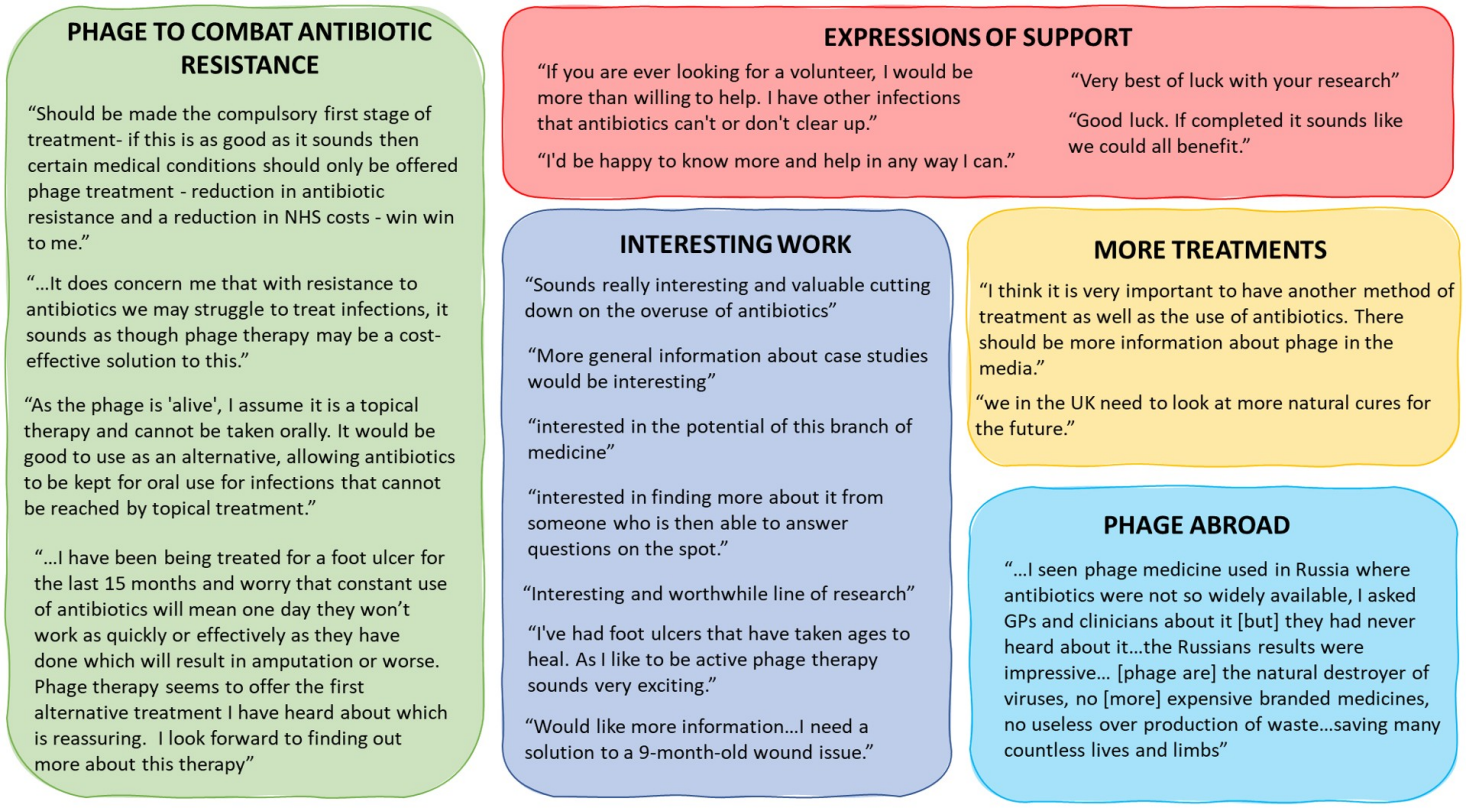
Further comments. 18 of 20 shown.

The final question in the survey gave patients an opportunity to express any further comments they might have. Of the 55 respondents, 20 provided further comments, 18/20 remarks are shown in Fig 4. Two comments were disregarded as uninterpretable, these were ‘dislike the numbness in my feet which varies. Feel it inhibits my balance’ and ‘don’t know what it is’.

Key themes among the comments were interest in phage therapy (n = 7), the ability of phage therapy to help combat AMR (n = 4), expressions of support (n = 4), demand for new treatments (n = 2) and patient impressions of phage therapy abroad (n = 1). One patient stated that phage therapy ‘should be made a compulsory first stage of treatment’ and another that ‘phage therapy seems to offer the first alternative therapy I have heard about which is reassuring’. Together these further comments indicated that patients were resoundingly supportive of phage therapy.

### Focus group findings

One focus group of five survey respondents was held as an opportunity to gain greater insight into patient thoughts and concerns about phage therapy. Questions and comments from the attendees could be divided into several broad themes, summarized in Table 3. Initial questions focused on an understanding and awareness of phage therapy. The discussion subsequently broadened out into comparisons between antibiotics and phage therapy, how phage therapy might work in practice and concerns about phage therapy. All the attendees were extremely supportive of phage therapy, and four of five strongly expressed a willingness to use phage therapy *in lieu* of intravenous antibiotics if possible, citing ease of use, the potential to not be admitted to hospital and the likely significantly reduced side effect profile. One attendee summed it up by commenting that ‘the NHS needs to be thinking outside the box when it comes to providing better treatment’.

**Table 3:** Key themes from the focus group (N = 5).

Theme	Example comments/questions
Lack of knowledge and awareness about phage therapy	• “What are phage?”
	• “How are [phage] different to antibiotics?”
	• “Where [are phage] used?”
	• “Have there been clinical trials and what were the results?”
Comparing phage therapy to antibiotics	• “Can antibiotics and phage be used together?”
	• “Would phage eventually replace IV antibiotics?”
	• “Could phage stop the infection getting bad enough that I need IV antibiotics in hospital?”
	• “There are concerns about lots of people not taking full courses of antibiotics because they feel better and don’t like taking tablets.”
Concerns about phage therapy	• “Why can phage not attack human cells?”
	• Can therapeutic phage mix with phage already in you?
	• “I would be concerned about mutations with the phage”
	• Does the production of phage involve animal testing?
	• “How do you prevent there being too many phage?”
	• “A basic leaflet in laymen’s terms would be good”
Practical questions about phage therapy	• “Does phage have to be given by injections? Can you drink it?”
	• “Is a blood test needed to find the right phage?”
	• “Is it best to identify things with a swab?”
	• “How long would you need to take it for?”
	• Phage therapy could be delivered at home
Support for phage therapy	• One patient had tried over the counter phage therapy seven years ago in Russia. It was used empirically orally and topically and there were no ill effects reported.
	• “Phage sounds like it’s too good to be true”
	• All patients were excited by the idea of phage therapy and strongly expressed a willingness to try phage, including instead of IV antibiotics if possible for 4/5 patients.
	• Perceived advantages were reduced side effects (compared to antibiotics) and reduced time in hospital.
	• “When will phage [therapy] start to be used in UK?”
	• It was suggested that phage therapy being perceived as ‘Russian’ might be a reason why it’s not used in the UK
	• “The NHS needs to be thinking outside the box when it comes to providing better treatment”

## Discussion

To the best of our knowledge this study represents the first investigation into what patients anywhere in the world think about the concept of phage therapy. To increase the impact of these results, we undertook this nationwide survey among a well-defined patient group. The diabetic foot patients that took part were both familiar with antibiotics and regularly engaged with the healthcare system. This patient group was chosen because phage therapy has been successfully used to treat diabetic foot infection in multiple contexts [18,19,22].

As well as engaging patients on the topic of phage therapy, this study briefly evaluated patients’ awareness of, and concern about, antibiotic resistance. There was a high level of awareness of antibiotic resistance and it’s causes among patients. Similar high levels of awareness of antibiotic resistance have been recorded in other contexts [23]. However, it was surprising to find that 7.3% of patients thought the cause was the human body becoming ‘immune’ to antibiotics. This has been encountered elsewhere [24], and highlights an important area for clarification in clinician-patient discussions. Arguably, such a high level of awareness and concern among this patient group reflects that diabetic foot patients are typically regular recipients of long courses of antibiotics for chronic infections. Nonetheless, it was encouraging that an overwhelming majority of patients felt that alternatives to antibiotics should be investigated.

Patients were largely aware of ‘viruses’, although the extent of conceptual understanding was not evaluated by this work. One focus group attendee said it would be helpful to have patient information that very simply explained key terms about infection, for instance exactly what a bacteria or virus is. Meanwhile, it seemed that over half of the patients that had heard of bacteriophage had done so outside the context of phage therapy. Just under 25% of 42 respondents had heard of ‘bacteriophage’ or ‘phage’, largely from media (mainly online) sources or their own education, while just 9.1% of 55 respondents had heard of phage therapy. A greater awareness of phage than phage therapy may reflect the wider importance of phage in basic and modern molecular biology. However, this discrepancy was surprising, given the increasing Western interest in phage therapy, and it may be that some patients were unable to recall whether or not they heard about phage in the context of therapy.

Overall patients were comparably concerned about the prospect of either phage or antibiotic therapy, with just 2% difference in overall concern expressed. However, the proportion of patients expressing moderate or extreme concern was less for phage than for antibiotics. This likely reflected patient’s earlier concerns about antibiotic resistance and, from the focus group, the side effect profile associated with intravenous antibiotics. Where concerns about phage therapy were expressed, they were largely focussed around safety, potential side effects and efficacy (vs. antibiotics).

Patient desire for information varied markedly, with one focus group attendee asking about why phage were not able to infect human cells. Because phage therapy is the use of a live virus to treat bacterial infection it will be important to reassure patients by clearly explaining the basic virology and differences between human and bacterial cells that underpin phage therapy. Such concerns are understandable, given how conceptually different phage therapy is to traditional Western medicine. This study suggests that strategies to mitigate patient concerns should include the provision of patient information materials, patient education and the education of healthcare staff delivering phage therapy to enable them to adequately address questions patients may have about their phage therapy.

There was a high level of patient acceptability of phage therapy. An overwhelming majority of patients would accept phage therapy if suggested by their doctor. Unsurprisingly, the majority of patients would try phage therapy as an alternative to amputation. Currently most Western phage therapy is undertaken on a compassionate basis, and evidently there would be patient demand for such an approach in the UK. Reassuringly, most patients expressed a willingness to participate in a clinical trial. Although there have been few clinical trials of phage therapy in the West [12,13,25], this question, unlike the last, explored patient responses to receiving phage therapy on an unpressured, non-critical, basis. There was also high demand from this patient group to see phage therapy introduced as an additional treatment option in Scotland, where there are approximately 27 major lower limb diabetic amputations every week [26]. Amputation is extremely costly to the patient, the NHS and wider society and arguably phage therapy has the potential to substantially mitigate these costs.

This study has several limitations. The survey response rate was lower than anticipated, and consequently the sample size represents just 0.4% of 14,059 diabetic foot patients in Scotland [26]. While this study has provided an invaluable insight into patient perceptions of phage therapy, the sample size limits extrapolation and statistical power. Moreover, the patient acceptability of phage therapy described in this study is limited to diabetic foot infection patients and acceptability of phage therapy may vary by clinical indication. The study also relied on self-selection, which may mean that patients self-motivated to find out more about health topics or participate in research were disproportionately represented. Additional selection bias may have arisen as patients with infections refractory to antibiotics may be disproportionately likely to seek, and be enthusiastic about, alternative therapies. As with any such study, there was also a small but inherent risk of confirmation bias. Practically undertaking the study online, with paper copies only in select clinics, presented difficulties for some patients. The study team were contacted four times by patients wanting to complete the survey but who were unable to due to lack of internet access. The identification of themes among patient’s free-text responses was open to investigator bias or misinterpretation, however this was mitigated by cross-checking with a second author.

Phage therapy may be decades away from clinical use, but these data encouragingly show exceptional levels of patient acceptability and a magnitude of concern comparable with antibiotics. It is important that healthcare professionals and policy makers realise that, if clearly explained, patients are likely to be very accepting of phage therapy in the West.

## Supporting information

S1 File A blank copy of the patient perception survey. (DOCX)

S2 File The survey participant information sheet and data protection information sheet. (DOCX)

S3 File The focus group participant information sheet, data protection information sheet and informed consent form. (DOCX)

S4 File Survey responses (anonymised minimal underlying dataset). (XLSX)

## Abbreviations

DFIs: diabetic foot infections
DFUs: diabetic foot ulcers
NHS: National Health Service
QEUH: Queen Elizabeth University Hospital
UK: United Kingdom

## References

[1] International Diabetes Federation. Diabetes facts & figures. [cited 3 Mar 2020]. https://www.idf.org/aboutdiabetes/what-is-diabetes/facts-figures.html

[2] Armstrong DG, Boulton AJM, Bus SA. Diabetic Foot Ulcers and Their Recurrence. N Engl J Med. 2017;376: 2367–2375. 10.1056/NEJMra1615439 28614678

[3] Prompers L, Huijberts M, Apelqvist J, Jude E, Piaggesi A, Bakker K, et al High prevalence of ischaemia, infection and serious comorbidity in patients with diabetic foot disease in Europe. Baseline results from the Eurodiale study. Diabetologia. 2007;50: 18–25. 10.1007/s00125-006-0491-1 17093942

[4] Tascini C, Piaggesi A, Tagliaferri E, Iacopi E, Fondelli S, Tedeschi A, et al Microbiology at first visit of moderate-to-severe diabetic foot infection with antimicrobial activity and a survey of quinolone monotherapy. Diabetes Res Clin Pract. 2011;94: 133–139. 10.1016/j.diabres.2011.07.017 21824673

[5] Stacey HJ, Clements CS, Welburn SC, Jones JD. The prevalence of methicillin-resistant Staphylococcus aureus among diabetic patients: a meta-analysis. Acta Diabetol. 2019 10.1007/s00592-019-01301-0 30955124 PMC6597605

[6] Eleftheriadou I, Tentolouris N, Argiana V, Jude E, Boulton AJ. Methicillin-resistant Staphylococcus aureus in diabetic foot infections. Drugs. 2010;70: 1785–1797. 10.2165/11538070-000000000-00000 20836573

[7] Henig O, Pogue JM, Cha R, Kilgore PE, Hayat U, Ja’ara M, et al Epidemiology of Diabetic Foot Infection in the Metro-Detroit Area With a Focus on Independent Predictors for Pathogens Resistant to Recommended Empiric Antimicrobial Therapy. Open Forum Infect Dis. 2018;5 10.1093/ofid/ofy245 30402532 PMC6215454

[8] Young MJ. Unpublished clinical audit data, Diabetic Foot Clinic, The Royal Infirmary of Edinburgh. 2020.

[9] Ray A, Malin D, Nicolau DP, Wiskirchen DE. Antibiotic Tissue Penetration in Diabetic Foot Infections. J Am Podiatr Med Assoc. 2015;105: 520–531. 10.7547/14-036.1 26667505

[10] Kortright KE, Chan BK, Koff JL, Turner PE. Phage Therapy: A Renewed Approach to Combat Antibiotic-Resistant Bacteria. Cell Host Microbe. 2019;25: 219–232. 10.1016/j.chom.2019.01.014 30763536

[11] Schooley RT, Biswas B, Gill JJ, Hernandez-Morales A, Lancaster J, Lessor L, et al Development and Use of Personalized Bacteriophage-Based Therapeutic Cocktails To Treat a Patient with a Disseminated Resistant Acinetobacter baumannii Infection. Antimicrob Agents Chemother. 2017;61: e00954–17. 10.1128/AAC.00954-17 28807909 PMC5610518

[12] Jault P, Leclerc T, Jennes S, Pirnay JP, Que Y-A, Resch G, et al Efficacy and tolerability of a cocktail of bacteriophages to treat burn wounds infected by Pseudomonas aeruginosa (PhagoBurn): a randomised, controlled, double-blind phase 1/2 trial. Lancet Infect Dis. 2019;19: 35–45. 10.1016/S1473-3099(18)30482-1 30292481

[13] Rhoads D d., Wolcott R d., Kuskowski M a., Wolcott B m., Ward L s., Sulakvelidze A. Bacteriophage therapy of venous leg ulcers in humans: results of a phase I safety trial. J Wound Care. 2009;18: 237–243. 10.12968/jowc.2009.18.6.42801 19661847

[14] Dedrick RM, Guerrero-Bustamante CA, Garlena RA, Russell DA, Ford K, Harris K, et al Engineered bacteriophages for treatment of a patient with a disseminated drug-resistant Mycobacterium abscessus. Nat Med. 2019;25: 730 10.1038/s41591-019-0437-z 31068712 PMC6557439

[15] Abedon ST, García P, Mullany P, Aminov R. Editorial: Phage Therapy: Past, Present and Future. Front Microbiol. 2017;8 10.3389/fmicb.2017.00981 28663740 PMC5471325

[16] Comeau AM, Hatfull GF, Krisch HM, Lindell D, Mann NH, Prangishvili D. Exploring the prokaryotic virosphere. Res Microbiol. 2008;159: 306–313. 10.1016/j.resmic.2008.05.001 18639443

[17] Morozova VV, Vlassov VV, Tikunova NV. Applications of Bacteriophages in the Treatment of Localized Infections in Humans. Front Microbiol. 2018;9 10.3389/fmicb.2018.01696 30116226 PMC6083058

[18] Fish R, Kutter E, Wheat G, Blasdel B, Kutateladze M, Kuhl S. Bacteriophage treatment of intransigent Diabetic toe ulcers: A case series. J Wound Care. 2016;25: S27–S33.26949862

[19] Patel DR, Bhartiya SK, Kumar R, Shukla VK, Nath G. Use of Customized Bacteriophages in the Treatment of Chronic Nonhealing Wounds: A Prospective Study. Int J Low Extrem Wounds. 2019; 1534734619881076. 10.1177/1534734619881076 31752578

[20] Gupta P, Singh HS, Shukla VK, Nath G, Bhartiya SK. Bacteriophage Therapy of Chronic Nonhealing Wound: Clinical Study. Int J Low Extrem Wounds. 2019;18: 171–175. 10.1177/1534734619835115 31081402

[21] Rogóż P, Amanatullah DF, Międzybrodzki R, Manasherob R, Tikunova NV, Weber-Dąbrowska B, et al Phage Therapy in Orthopaedic Implant-Associated Infections. Phage Ther Pract Approach. 2019; 189–211. 10.1007/978-3-030-26736-0_8

[22] Weber-Dabrowska B, Mulczyk M, Gorski A. Bacteriophage therapy for infections in cancer patients. Clin Appl Immunol Rev. 2001;1: 131–134.

[23] Goettsche LS, Weig EA, Chung J, Hoff BM, Ince D, Wanat KA. Patient perceptions of antibiotic use and resistance at a single university dermatology clinic. J Dermatol Treat. 2019;30: 92–95. 10.1080/09546634.2018.1473549 29726725

[24] Brookes-Howell L, Elwyn G, Hood K, Wood F, Cooper L, Goossens H, et al ‘The Body Gets Used to Them’: Patients’ Interpretations of Antibiotic Resistance and the Implications for Containment Strategies. J Gen Intern Med. 2012;27: 766–772. 10.1007/s11606-011-1916-1 22065334 PMC3378752

[25] Wright A, Hawkins CH, Änggård EE, Harper DR. A controlled clinical trial of a therapeutic bacteriophage preparation in chronic otitis due to antibiotic-resistant Pseudomonas aeruginosa; a preliminary report of efficacy. Clin Otolaryngol. 2009;34: 349–357. 10.1111/j.1749-4486.2009.01973.x 19673983

[26] Scottish Diabetes Survey Monitoring Group. Scottish Diabetes Survey 2018. 2018. https://www.diabetesinscotland.org.uk/wp-content/uploads/2019/12/Scottish-Diabetes-Survey-2018.pdf

