# Supplementary material for: Patient perceptions of phage therapy for diabetic foot infection
Source: PLoS One. 2020 Dec 14;15(12):e0243947. doi: 10.1371/journal.pone.0243947 (PMC7735629; doi:10.1371/journal.pone.0243947)
Supplement: S1 File — (DOCX) [file pone.0243947.s001.docx]

SURVEY ID NUMBER:

___________________

Finding out what diabetic foot infection (DFI) patients think about phage therapy

This study is a patient survey.

This survey aims to find out:

1. Your awareness of antibiotic resistance
2. Your awareness of something called ‘phage therapy’
3. Concerns you might have about phage therapy
4. If you would consider having phage therapy

Don’t worry if you haven’t heard of antibiotics or phage therapy.

Any responses you give will be very useful.

There are five parts to this survey.

We think this survey should take **about 20 minutes to complete**.

This survey is being managed by Dr Josh Jones (University of Edinburgh). If you’ve any questions please contact him on:

[josh.jones@ed.ac.uk](mailto:josh.jones@ed.ac.uk) or XXXXXXXXXXX.

***Returning the survey:***

**Online:** it automatically comes back to us

**In person:** if you got this survey at a clinic please hand it to a member of clinic staff

**Part one: about you**

1) Please tell us where you got the survey from:

🞎 Via the **Scottish Diabetes Research Network**

🞎 At the **Edinburgh Royal Infirmary** foot clinic

🞎 At the **Queen Elizabeth Hospital (Glasgow)** foot clinic

2) You must only do this survey once. If you get offered it again, please refuse. Tick to confirm you have only done this copy.

🞎

3) How old are you?

_____ years

4) Gender:

🞎 Male 🞎 Female

5) Have you ever taken antibiotics for any illness?

🞎 Yes 🞎 No 🞎 Don’t know

6) How often have you taken a course of antibiotics in the last 12 months for any illness?

_____ times

7) How many separate times have you been an outpatient or inpatient for any reason in the last 12 months?

Outpatient _____ times in the last 12 months

Inpatient _____ times in the last 12 months

**Part two: antibiotics**

*Key information - please read before continuing*

- Bacteria can cause infections in people
- Antibiotics can be used to treat these infections
- Bacteria can become resistant to antibiotics, which means
  antibiotics no longer work

8) Before this survey, had you heard of ‘antibiotic resistance’?

🞎 Yes 🞎 No

a) If you had heard of ‘antibiotic resistance’ before this survey, please write where you heard about it.

b) If you have heard of ‘antibiotic resistance’, please write what you think causes antibiotic resistance.

9) How concerned are you about antibiotic resistance?

*Circle your response below.*

Extremely concerned

Not concerned

Slightly concerned

Moderately concerned

10) How much do you agree with this statement?

*‘Enough is being done to tackle antibiotic resistance.’*

*Circle your response below.*

Strongly agree

Agree

Neither agree or disagree

Don’t know

Strongly disagree

Disagree

a) If you think more should be done to tackle antibiotic resistance, please write what you think should be done.

11) Do you think alternatives to antibiotics should be investigated?

🞎 Yes 🞎 No

**Part three: have you heard of ‘phage’?**

12) Have you heard of viruses?

🞎 Yes 🞎 No 🞎 Not sure

a) If yes, have you heard of viruses that kill bacteria, also known as ‘bacteriophage’ or ‘phage’?

🞎 Yes 🞎 No 🞎 Not sure

b) If you have heard of ‘bacteriophage’ or ‘phage’, please write where you heard about them.

13) Have you heard of ‘phage’ or ‘bacteriophage’ therapy?

🞎 Yes 🞎 No 🞎 Not sure

a) If yes, please write where you heard about ‘phage’ or ‘bacteriophage’ therapy.

**Part four: what you think about phage therapy**

*Key information - please read before continuing*

- Phage are viruses (i.e. living things) that kill bacteria
- Phage don’t infect or harm people
- Phage are the most common living things on the planet and found almost everywhere
- Phage can be used instead of antibiotics to treat infections in people
- Phage are already used to kill bacteria in agriculture and food preparation

14) How concerned are you about being treated with antibiotics?
*Circle your response below.*

Extremely concerned

Moderately concerned

Slightly concerned

Not concerned

15) Having read the key information above, how concerned would you be about being treated using a phage instead of an antibiotic?
*Circle your response below.*

Extremely concerned

Moderately concerned

Slightly concerned

Not concerned

16) If there was no antibiotic treatment available for your infection would you consider phage therapy?

🞎 Yes 🞎 No 🞎 Not sure

17) Based on what you already know and/or the information at the start of this section, please write any concerns you would have about being treated with phage therapy.

a) What, if anything, could be done to reduce your concerns?

*DEBRIEF – **there are more questions after***

**What are bacteriophage (phage)?**

- Phage were discovered in 1915
- The term ‘bacteriophage’ literally means ‘bacteria eater’
- Phage are natural viruses that can kill bacteria
- There’s roughly 10 times as many phage on/in us as the number of bacteria on/in us, that’s 100 times the number cells in the body
- Phage are the most abundant ‘living’ thing on the planet – they are found wherever there are bacteria
- Phage don’t infect or harm people
- Unlike antibiotics, phage are living things that kill bacteria, they are very fussy about the bacteria they kill

**The history of phage therapy**

- Phage have been used to treat infections since their discovery
- Early phage therapy studies weren’t all done well and the discovery of antibiotics in Western Europe meant phage therapy fell out of fashion in the West
- Phage therapy continues in Eastern Europe and trials are underway in Western Europe
- In America phage are used to protect food from bacteria

**How does phage therapy work?**

- Most phage used for therapy are natural
- It’s easy to put onto the skin or wounds
  - A 2016 study in America successfully treated foot ulcers by dripping a small amount of liquid containing phage onto the ulcer once a week
- Studies so far have shown phage therapy to be very safe, with few or no side effects

**Challenges**

- There are regulatory challenges to phage therapy in the UK
- Phage therapy only works if the phage used can kill the bacteria causing the infection
- Natural phage cannot be patented, are easy to use and can be easily copied, making phage financially unattractive to companies who develop drugs

**Part five: what you think about phage therapy after reading the debrief**

18) Having read the debrief, if your doctor recommended phage therapy, would you agree?

🞎 Yes 🞎 No 🞎 Not sure

1. If not sure, please say why:

19) Having read the debrief, if you had an infection for which the only treatment left was amputation, would you try phage therapy first?

🞎 Yes 🞎 No 🞎 Not sure

1. If not sure, please say why:

20) Having read the debrief, if there was a clinical trial for phage therapy, would you be willing to take part?

🞎 Yes 🞎 No 🞎 Not sure

1. If not sure, please say why:

21) Would you like to see phage therapy as an additional treatment option for diabetic foot infections in Scotland?

🞎 Yes 🞎 No 🞎 Not sure

1. If not sure, please say why:

22) Please give us an e-mail address if you’d like to get the results of this study.

*Your details will only be used to send you the results and will be deleted afterwards, please see the cover sheet for details.*

________________________________________________________

23) Please write any further comments you have about phage therapy.

Thank you for taking the time to help, we really appreciate it!

Please follow the instructions on the front page to make sure your survey finds its way back to us.
