# Supplementary material for: Patient perceptions of phage therapy for diabetic foot infection
Source: PLoS One. 2020 Dec 14;15(12):e0243947. doi: 10.1371/journal.pone.0243947 (PMC7735629; doi:10.1371/journal.pone.0243947)
Supplement: S2 File — (DOCX) [file pone.0243947.s002.docx]

**GUIDANCE**

**Participant Information Sheet**

**Finding out what diabetic foot infection (DFI) patients think about phage therapy**

**We’re inviting you to take part in a research study. This study is a questionnaire. Before you decide whether or not to take part, it’s important for you to understand why the research is being done and what it will involve. Please take time to read the following information carefully. Talk to others about the study if you want. Contact us if anything isn’t clear, or if you’d like more information. Take time to decide whether or not you want to take part.**

| **What is the study for?** |
| --- |
| We can get infections from bacteria. Antibiotics can treat them – but bacteria can become resistant to antibiotics, so they no longer kill the bacteria.  Because the number of resistant bacteria is going up, researchers are investigating other ways of killing bacteria.  Bacteriophage (phage) look promising. They are viruses that kill bacteria. They are the most common living things on the planet and found almost everywhere.  Phage can be used instead of antibiotics to treat human infections – it’s called ‘phage therapy’. In the UK doctors don’t yet use phage therapy to treat people, but industry uses phage control bacteria in agriculture and food preparation.  We want to find out just how aware diabetic foot infection patients are of bacterial resistance to antibiotics – and how they would feel about getting phage therapy. We hope our results will supply the evidence needed to back investment in phage therapy research in Scotland.  We’re inviting past and present diabetic foot infection patients to take part. |
| **Why have I been invited to take part?** |
| Because you have a past or present diagnosis of diabetic foot infection. |
| **Do I have to take part?** |
| No, it’s up to you. If you do decide to take part, you keep this information sheet. You can leave the study at any time, without giving a reason. Deciding not to take part or leaving the study won’t affect the healthcare that you get, or your legal rights. |
| **What will happen if I take part?** |
| We’ll ask you to complete this anonymous short survey at a time and place that suits you.  You can access the survey on the internet at [LINK]. Your survey responses won’t be sent to us until you’ve clicked the ‘Finish’ button at the end.  You might have a paper copy if you are completing the survey in a clinic, and you can hand the survey into the clinic reception desk.  We’re not paying any expenses. |
| **Is there anything I need to do or avoid?** |
| No. |
| **What are the possible benefits of taking part?** |
| There are no direct benefits to you taking part in this study, but the results from it might help to improve the healthcare patients get in the future. |
| **What are the possible disadvantages of taking part?** |
| We think the survey will take you 15-20 minutes to do.  Taking part won’t affect any insurance you have. |
| **What if there are any problems?** |
| If you have a concern about any aspect of this study please contact Dr Josh Jones ([josh.jones@ed.ac.uk](mailto:josh.jones@ed.ac.uk)) who will do his best to answer your questions.  In the unlikely event that something goes wrong and you are harmed during the research and this is due to someone’s negligence then you may have grounds for a legal action for compensation against NHS Scotland but you may have to pay your legal costs. You can still use the normal National Health Service complaints procedures (if appropriate). |
| **What will happen if I don’t want to carry on with the study** |
| You can withdraw your consent to take part in this survey at any time. You’re under no obligation to return your survey.  Note though, because the survey is anonymous, after you have returned your survey we can’t remove your data from the study. |
| **What happens when the study is finished?** |
| We’ll analyse the responses we get. Our analysis may form the basis of a scientific publication that also includes direct quotation of individual respondents’ responses to free-text questions.  We won’t pass the survey responses on to third parties. |
| **Will my taking part be kept confidential?** |
| This survey is anonymous.  We’ll keep all the information we collect during the course of the research confidential and we’ll meet the strict laws which safeguard your privacy at every stage.  Data about you will not be shared beyond the UK or European Economic Area.  Survey responses will be kept by Dr Josh Jones at the University of Edinburgh in line with data protection laws. |
| **What will happen to the results of the study?** |
| We’ll write up the study for publication in an open access journal. It will be available right across the world.  You won’t be identified in any published results |
| **Who is organising and funding the research?** |
| Dr Josh Jones (University of Edinburgh) has organised this study and the University of Edinburgh and NHS Lothian have co-sponsored it.  No funding is required for this study. |
| **Who has reviewed the study?** |
| The Scottish Diabetes Research Network Public and Patient Involvement group commented on the preparation of our study.  All research in the NHS is looked at by an independent group of people called a Research Ethics Committee. London - Surrey Borders Research Ethics Committee says the study is ethical. NHS management and the relevant NHS Research and Development departments has also approved it. |
| **Researcher Contact Details** |
| If you have any more questions about the study please contact Dr Josh Jones (University of Edinburgh) on XXXX or [josh.jones@ed.ac.uk](mailto:josh.jones@ed.ac.uk). |
| **Independent Contact Details** |
| If you would like to discuss this study with someone independent of the study please contact XXXX (NHS Research Scotland Diabetes Network) on XXXX. |
| **Complaints** |
| If you want to make a complaint about the study please contact the NHS Lothian patient experience team:  Patient Experience Team  2 – 4 Waterloo Place, Edinburgh, EH1 3EG  [feedback@nhslothian.scot.nhs.uk](mailto:feedback@nhslothian.scot.nhs.uk)  0131 536 3370 |

# **Data Protection Information Sheet**

**Finding out what diabetic foot infection (DFI) patients think
about phage therapy**

The EU General Data Protection Regulation (GDPR), along with the UK Data Protection Act, governs the processing (holding or use) of personal data in the UK.

You are receiving this as you are considering being a participant on this clinical research study. The information below details what data will be held about you and who will hold or store this.

University of Edinburgh and NHS Lothian are the co-sponsors for this study based in the United Kingdom. We will use information from you and/or your medical records in order to undertake this study and will act as the data controller for this study. This means that we are responsible for looking after your information and using it properly. The co-sponsors will keep identifiable information about you for 6 months after the study has finished.

As a University/NHS organisation we use personally-identifiable information to conduct research to improve health, care and services. As a publicly-funded organisation, we have to ensure that it is in the public interest when we use personally-identifiable information from people who have agreed to take part in research.  This means that when you agree to take part in a research study, we will use your data in the ways needed to conduct and analyse the research study. Your rights to access, change or move your information are limited, as we need to manage your information in specific ways in order for the research to be reliable and accurate. If you withdraw from the study, we will keep the information about you that we have already obtained. To safeguard your rights, we will use the minimum personally-identifiable information possible.

| **Providing personal data directly e.g. verbally, in a questionnaire or from your care provider** |
| --- |

The University will use your e-mail address (survey respondents only) to contact you with the results of the research study. The University/NHS will record your name for focus group consent and booking only. Individuals from either sponsoring organisation stated above and regulatory organisations may look at your medical and research records to check the accuracy of the research study. The University will pass these details to NHS Lothian along with the anonymous information collected from you. The only people in The University or NHS Lothian who will have access to information that identifies you will be people who need to contact you to send you the survey results or book you a place at, or take your consent to participate in, a focus group. Staff in The University or NHS Lothian may audit the data collection process. The people who analyse the survey responses you give will not be able to identify you and will not be able to find out your name or contact details.

The University will keep identifiable information about you from this study for 6 months after the study has finished.

| **Contact for further information** |
| --- |

You can find out more about how we use your information and our legal basis for doing so in our Privacy Notice at [www.accord.scot](http://www.accord.scot).

For further information on the use of personal data by NHS sites, please link to the Health Research Authority (HRA) website; <https://www.hra.nhs.uk/information-about-patients/>.

If you wish to raise a complaint on how we have handled your personal data, you can contact our Data Protection Officer who will investigate the matter. If you are not satisfied with our response or believe we are processing your personal data in a way that is not lawful you can complain to the Information Commissioner’s Office (ICO) at <https://ico.org.uk/>.

Data Protection Officer contact information:

| **University of Edinburgh**  Data Protection Officer  Governance and Strategic Planning  University of Edinburgh  Old College  Edinburgh  EH8 9YL  Tel: 0131 651 4114  [dpo@ed.ac.uk](mailto:dpo@ed.ac.uk) | **NHS Lothian**  Data Protection Officer  NHS Lothian  Waverley Gate  2-4 Waterloo Place  Edinburgh  EH1 3EG  Tel: 0131 465 5444  [Lothian.DPO@nhs.net](mailto:Lothian.DPO@nhs.net) |
| --- | --- |
