# Supplementary material for: Patient perceptions of phage therapy for diabetic foot infection
Source: PLoS One. 2020 Dec 14;15(12):e0243947. doi: 10.1371/journal.pone.0243947 (PMC7735629; doi:10.1371/journal.pone.0243947)
Supplement: S3 File — (DOCX) [file pone.0243947.s003.docx]

**GUIDANCE**

**Participant Information Sheet**

**Finding out what diabetic foot infection (DFI) patients think about phage therapy (focus groups)**

**We’re inviting you to take part in a research study. The study involves a small group of patients discussing their thoughts with a few researchers (focus groups). Before you decide whether or not to take part, it’s important for you to understand why the research is being done and what it will involve. Please take time to read the following information carefully. Talk to others about the study if you want. Contact us if anything isn’t clear, or if you’d like more information. Take time to decide whether or not you want to take part.**

| **What is the study for?** |
| --- |
| This study is an extension of the survey that you have just completed. It offers the opportunity to meet with a researcher and other diabetic foot patients to talk a bit more about your thoughts about phage therapy.  We’re only inviting survey respondents to participate. |
| **Why have I been invited to take part?** |
| Because you have a past or present diagnosis of diabetic foot infection AND have completed the survey ‘finding out what diabetic foot infection (DFI) patients think about phage therapy’. |
| **Do I have to take part?** |
| No, it’s up to you. If you do decide to take part, you keep this information sheet. You can leave the study at any time, without giving a reason. Deciding not to take part or leaving the study won’t affect the healthcare that you get, or your legal rights.  You can take your time to think about whether you would like to take part. If you then decide you want to take part please contact Dr Josh Jones ([josh.jones@ed.ac.uk](mailto:josh.jones@ed.ac.uk), XXXX) so that we can book you a space. The only information we need for booking is your name and a phone number or e-mail address so that we can contact you if the focus group is cancelled or rearranged. |
| **What will happen if I take part?** |
| You can look at the dates, times and places where focus groups will be held.  There will be four times available, two at the Edinburgh Royal Infirmary and two at the Queen Elizabeth hospital in Glasgow.  You can choose which one you want to go to. There will be no more than 7 patients per group and two researchers or members of clinical staff.  On the day you will be able to discuss what you think about phage therapy with the researchers and other patients. The researchers will offer ideas of topics to talk about, but it’ll be informal and relaxed. Tea and coffee will be provided.  The session will not be recorded, but the researchers will take notes. No personal information will be held in the notes and you can ask to see them if you want to.  It’s up to you to decide whether or not to book a space. If you’d like to take part please contact Dr Jones to book a place. You can take as long as you like to decide.  We aren’t able to provide travel expenses. |
| **Is there anything I need to do or avoid?** |
| No. |
| **What are the possible benefits of taking part?** |
| There are no direct benefits to you taking part in this study, but the results from it might help to improve the healthcare patients get in the future. |
| **What are the possible disadvantages of taking part?** |
| The focus group will take no more than 60 minutes.  Taking part won’t affect any insurance you have. |
| **What if there are any problems?** |
| If you have a concern about any aspect of this study please contact Dr Josh Jones ([josh.jones@ed.ac.uk](mailto:josh.jones@ed.ac.uk)) who will do his best to answer your questions.  In the unlikely event that something goes wrong and you are harmed during the research and this is due to someone’s negligence then you may have grounds for a legal action for compensation against NHS Scotland but you may have to pay your legal costs. You can still use the normal National Health Service complaints procedures (if appropriate). |
| **What will happen if I don’t want to carry on with the study** |
| You can withdraw your consent to take part at any time. You’re under no obligation to stay for the whole discussion. |
| **What happens when the study is finished?** |
| We’ll look at the notes from each focus group. Our analysis may form the basis of a scientific publication that also includes direct anonymous quotation of individual respondents’ responses.  We won’t pass our notes on to third parties. |
| **Will my taking part be kept confidential?** |
| Your taking part will not be shared, but you will meet researchers and other patients.  We will need your name and a form of contact (phone number or e-mail address) for booking you a space and to contact you in the unlikely event the group is cancelled or rearranged.  We’ll keep all the information we collect during the course of the research confidential and we’ll meet the strict laws which safeguard your privacy at every stage.  Data will be kept by Dr Josh Jones at the University of Edinburgh in line with data protection laws. |
| **What will happen to the results of the study?** |
| We’ll write up the study for publication in an open access journal. It will be available right across the world.  You won’t be identified in any published results |
| **Who is organising and funding the research?** |
| Dr Josh Jones (University of Edinburgh) has organised this study and the University of Edinburgh and NHS Lothian have co-sponsored it.  No funding is required for this study. |
| **Who has reviewed the study?** |
| Public and Patient Involvement groups from Dundee and Edinburgh commented on the preparation of our study.  All research in the NHS is looked at by an independent group of people called a Research Ethics Committee. London - Surrey Borders Research Ethics Committee says the study is ethical. NHS management has also approved it. |
| **Researcher Contact Details** |
| If you have any more questions about the study please contact Dr Josh Jones (University of Edinburgh) on 0131 650 4570 or [josh.jones@ed.ac.uk](mailto:josh.jones@ed.ac.uk). |
| **Independent Contact Details** |
| If you would like to discuss this study with someone independent of the study please contact XXXX (NHS Research Scotland Diabetes Network) on XXXX. |
| **Complaints** |
| If you want to make a complaint about the study please contact the NHS Lothian patient experience team:  Patient Experience Team  2 – 4 Waterloo Place, Edinburgh, EH1 3EG  [feedback@nhslothian.scot.nhs.uk](mailto:feedback@nhslothian.scot.nhs.uk)  0131 536 3370  If you wish to raise a complaint on how we have handled your personal data, you can contact our Data Protection Officer who will investigate the matter. If you are not satisfied with our response or believe we are processing your personal data in a way that is not lawful you can complain to the Information Commissioner’s Office (ICO) at <https://ico.org.uk/>.  Data Protection Officer contact information:   \| University of Edinburgh Data Protection Officer  Governance and Strategic Planning  University of Edinburgh  Old College  Edinburgh  EH8 9YL  Tel: 0131 651 4114  [dpo@ed.ac.uk](mailto:dpo@ed.ac.uk) \| NHS Lothian Data Protection Officer  NHS Lothian  Waverley Gate 2-4  Waterloo Place  Edinburgh  EH1 3EG  Tel: 0131 465 5444 [Lothian.DPO@nhs.net](mailto:Lothian.DPO@nhs.net) \| \| --- \| --- \| |

# **Data Protection Information Sheet**

**Finding out what diabetic foot infection (DFI) patients think
about phage therapy (focus groups)**

The EU General Data Protection Regulation (GDPR), along with the UK Data Protection Act, governs the processing (holding or use) of personal data in the UK.

You are receiving this as you are considering being a participant on this clinical research study. The information below details what data will be held about you and who will hold or store this.

University of Edinburgh and NHS Lothian are the co-sponsors for this study based in the United Kingdom. We will use information from you and/or your medical records in order to undertake this study and will act as the data controller for this study. This means that we are responsible for looking after your information and using it properly. The co-sponsors will keep identifiable information about you for 6 months after the study has finished.

As a University/NHS organisation we use personally-identifiable information to conduct research to improve health, care and services. As a publicly-funded organisation, we have to ensure that it is in the public interest when we use personally-identifiable information from people who have agreed to take part in research.  This means that when you agree to take part in a research study, we will use your data in the ways needed to conduct and analyse the research study. Your rights to access, change or move your information are limited, as we need to manage your information in specific ways in order for the research to be reliable and accurate. If you withdraw from the study, we will keep the information about you that we have already obtained. To safeguard your rights, we will use the minimum personally-identifiable information possible.

| **Providing personal data directly e.g. verbally, in a questionnaire or from your care provider** |
| --- |

The University will use your phone number and/or e-mail address to book you a place at a focus group and tell you if it’s cancelled or rearranged. Individuals from either sponsoring organisation stated above and regulatory organisations may look at your research records to check the accuracy of the research study. The University will pass these details to NHS Lothian along with the anonymous information collected from you. The data you provide will be held securely by Dr Jones and only used it to book you a place at a focus group and inform you if that group is cancelled or rearranged. Staff in The University or NHS Lothian may audit the data collection process. The people who analyse the survey responses you give will not be able to identify you and will not be able to find out your name or contact details.

The University will keep identifiable information about you from this study for 6 months after the study has finished.

| **Contact for further information** |
| --- |

You can find out more about how we use your information and our legal basis for doing so in our Privacy Notice at [www.accord.scot](http://www.accord.scot).

For further information on the use of personal data by NHS sites, please link to the Health Research Authority (HRA) website; <https://www.hra.nhs.uk/information-about-patients/>.

If you wish to raise a complaint on how we have handled your personal data, you can contact our Data Protection Officer who will investigate the matter. If you are not satisfied with our response or believe we are processing your personal data in a way that is not lawful you can complain to the Information Commissioner’s Office (ICO) at <https://ico.org.uk/>.

Data Protection Officer contact information:

| **University of Edinburgh**  Data Protection Officer  Governance and Strategic Planning  University of Edinburgh  Old College  Edinburgh  EH8 9YL  Tel: 0131 651 4114  [dpo@ed.ac.uk](mailto:dpo@ed.ac.uk) | **NHS Lothian**  Data Protection Officer  NHS Lothian  Waverley Gate  2-4 Waterloo Place  Edinburgh  EH1 3EG  Tel: 0131 465 5444  [Lothian.DPO@nhs.net](mailto:Lothian.DPO@nhs.net) |
| --- | --- |

**CONSENT FORM**

Finding out what diabetic foot infection (DFI) patients
think about phage therapy (focus groups)

|  | | Please **initial** box |
| --- | --- | --- |
|  | 1. I confirm that I have read and understand the information sheet (02 SEPT 2019 and Version 3.0) for the above study. I have had the opportunity to consider the information, ask questions and have had these questions answered satisfactorily. | ⬜ |
|  | 1. I understand that my participation is voluntary and that I am free to withdraw at any time without giving any reason and without my medical care and/or legal rights being affected. | ⬜ |
|  | 1. I have completed the survey ‘finding out what diabetic foot infection (DFI) patients think about phage therapy’ | ⬜ |
|  | 1. I agree to take part in the above study | ⬜ |

|  |  |  |  |  |
| --- | --- | --- | --- | --- |
| Name of Person Giving Consent |  | Date |  | Signature |
|  |  |  |  |  |
| Name of Person Receiving Consent |  | Date |  | Signature |

1x original – into Site File; 1x copy – to Participant
